# The MEK/ERK Pathway Promotes NOTCH Signalling in Pancreatic Cancer Cells

**DOI:** 10.1371/journal.pone.0085502

**Published:** 2013-12-31

**Authors:** Isabelle Tremblay, Emanuel Paré, Dominique Arsenault, Mélanie Douziech, Marie-Josée Boucher

**Affiliations:** Department of Medicine, Faculté de Médecine et des Sciences de la Santé, Université de Sherbrooke, Sherbrooke, Québec, Canada; Columbia University, United States of America

## Abstract

Activation of the NOTCH receptors relies on their intracellular proteolysis by the gamma-secretase complex. This cleavage liberates the NOTCH intracellular domain (NIC) thereby allowing the translocation of NIC towards the nucleus to assemble into a transcriptional platform. Little information is available regarding the regulatory steps operating on NIC following its release from the transmembrane receptor up to its association with transcriptional partners. Interfering with these regulatory steps might potentially influences the nuclear outcome of NOTCH signalling. Herein, we exploited a reliable model to study the molecular events occurring subsequent to NOTCH1 cleavage. In pancreatic cancer cells, pulse of NOTCH1 activation led to increased expression of NOTCH target genes namely HES1 and c-MYC. We uncovered that, upon its release, the NOTCH1 intracellular domain, NIC1, undergoes a series of post-translational modifications that include phosphorylation. Most interestingly, we found that activation of the MEK/ERK pathway promotes HES1 expression. Inhibition of the gamma-secretase complex prevented the MEK/ERK-induced HES1 expression suggesting a NOTCH-dependent mechanism. Finally, higher levels of NIC1 were found associated with its transcriptional partners [CBF1, Su(H) and LAG-1] (CSL) and MASTERMIND-LIKE 1 (MAML1) upon MEK/ERK activation providing a potential mechanism whereby the MEK/ERK pathway promotes expression of NOTCH target genes. For the first time, our data exposed a signalling pathway, namely the MEK/ERK pathway that positively impacts on NOTCH nuclear outcome.

## Introduction

 The NOTCH receptors orchestrate a number of developmental processes besides ensuring adult tissue homeostasis [1,2]. This highly conserved signalling pathway has a relatively simple molecular architecture. Upon ligand binding, the transmembrane NOTCH receptors (NOTCH 1-4) undergo sequential cleavages by ADAM-metalloproteases and the gamma-secretase complex. The latter, blocked by gamma-secretase inhibitors, releases the NOTCH intracellular domain (NIC) that is free to translocate towards the nucleus to collaborate with the DNA-binding protein [CBF1, Su(H) and LAG-1] (CSL) and the co-activator MASTERMIND-LIKE 1 (MAML1) to modulate gene expression. The best-characterized target genes of the NOTCH pathway are certainly members of the HAIRY ENHANCER OF SPLIT (HES) family, themselves regulators of transcription [1–3]. One distinct characteristic of the NOTCH signalling pathway is thus the dual role of the receptor i.e. sensing the signal and achieving the response. Little is known about the regulatory steps operating on NIC following its release from the transmembrane receptor to its transcriptional action. However, the nuclear outcome of NOTCH signalling is, most likely, tightly controlled in order to ensure the precise regulation of signal strength and duration. Further studies are thus clearly needed to unravel the mechanisms by which the cleaved receptor coordinates gene expression. In addition, identification of potential modulator of NOTCH signalling should improve our understanding of this apparent simplistic pathway.

 Aberrant NOTCH signalling was shown to play important roles in haematological malignancies [4] and some solid tumours [2] such as pancreatic ductal adenocarcinoma (PDA). Indeed, reactivation of NOTCH signalling is observed early in PDA pathogenesis and persists throughout the progression of the disease [5–8]. Exome sequencing of human PDA tissues provided further support of a critical role for NOTCH signalling in pancreatic carcinogenesis [9]. Interestingly, blockade of NOTCH signalling with gamma-secretase inhibitor prevented the progression of premalignant pancreatic lesions to PDA in a mouse model of KRAS-induced PDA [10,11]. Noteworthy, KRAS downstream signalling plays critical role in pancreatic carcinogenesis as oncogenic mutation in KRAS are found in 95% of PDA [9,12]. Furthermore, reduced NOTCH signalling in human pancreatic cancer cell lines correlated with reduced proliferation rates, increased apoptosis, decreased anchorage-independent growth and decreased invasion properties [11,13–16]. This connection between NOTCH and RAS signalling is not unique to pancreatic cancer cells. Indeed, RAS and NOTCH signalling were shown to cooperate in promoting carcinogenesis in breast cancer cells, melanoma and leukemia [17–19]. Globally, targeting NOTCH signalling appears an attractive new therapeutic strategy particularly for PDA patients [20]. However, a better understanding of the pathway is critical in order to develop effective NOTCH inhibitors and/or antagonists since gamma-secretase inhibitors, although useful, are not NOTCH specific and indiscriminately impact all signalling pathways regulated by the gamma-secretase complex besides instigating gastrointestinal toxicity [21–23]. 

 In this study, we exploited a reliable model to study the molecular events occurring after the cleavage of the transmembrane NOTCH1 receptor up to the nuclear localization of the cleaved NOTCH1 fragment (NIC1). We uncovered that, upon its release, NIC1 undergoes hierarchical phosphorylation in pancreatic cancer cells that correlates with expression of NOTCH target genes such as HES1. Most interestingly, we found that activation of the MEK/ERK pathway promotes HES1 expression through NOTCH-dependent mechanisms. 

## Materials and Methods

### Cell Culture and NOTCH Activation Procedure

The HEK293T and the human pancreatic cancer cell lines MIA PaCa-2 and BxPC-3 were obtained from ATCC and cultured as previously described [24]. 

To induce a pulse of NOTCH activation, we added ethylene glycol-bis(2-aminoethylether)-*N,N,N*’,*N’-* tetraacetic acid (EGTA) (4mM) for 15 minutes to exponentially growing MIA PaCa-2 cells. EGTA was then removed by replacing the media with fresh normal culture media (DMEM).

### Antibodies and Reagents

The specific antibody recognizing only the cleaved NOTCH1 (D3B8) (NIC1) was obtained from Cell Signaling. Antibodies against dual-phosphorylated (active) ERK1/2 (pERK1/2), CSL, MAML1 and GAPDH were purchased from Cell Signaling. HES1 antibody was from Abcam. Antibody for the detection of total ERK1/2 was from Santa Cruz. MYC and HA antibodies were obtained from Roche. The gamma-secretase inhibitor N-[N-(3,5-Difluorophenacetyl-L-alanyl)]-S-phenylglycine *t*-Butyl Ester (DAPT) and the MEK1/2 inhibitor U0126 were purchased from Calbiochem. All other materials were from Sigma unless stated otherwise. 

### Western Blot

As previously published [24], cells were lysed in Triton buffer (1% Triton X-100, 50mM Tris pH 7.5, 100mM NaCl, 5mM EDTA, 0.2mM orthovanadate, 40mM β-glycerophosphate, 50mM sodium fluoride, 10% glycerol, 1mM PMSF, 0.5μg/ml leupeptin, 1μg/ml pepstatin and 0.5μg/ml aprotinin) and cleared of cellular debris by centrifugation. Equal amount of proteins were separated by SDS-PAGE (polyacrylamide gel electrophoresis) and proteins were detected immunologically after electrotransfer onto nitrocellulose membranes. 

### Transient Transfections

Experiments were performed as described previously [24–26]. Briefly, HEK293T cells were transfected with Lipofectamine 2000 (Invitrogen) according to the manufacturer’s recommendations. The vector encoding a MYC-tagged version of the cytoplasmic domain of mouse *notch1* (pLIA-NIC1) was obtained from Addgene (plasmid 15131). HA-tagged wild-type MEK1 (MEK1 WT) and constitutively active mutant of MEK1 (MEK1 CA)-encoding cDNAs were kindly provided by N. Rivard (Université de Sherbrooke, Canada) and were previously described [27]. Cells were lysed twenty-four hours post-transfection and prepared for western blot. 

### Immunoprecipitation, Phosphatase and Kinase assays

Immunoprecipitations were essentially performed as previously described [25,26]. Briefly, cells were washed twice with ice-cold PBS, lysed in Triton buffer and cleared of cellular debris by centrifugation. Primary antibody was added to 2mg of cleared cell lysates and incubated for 3h at 4°C under agitation. Endogenous NIC1 was immunoprecipitated using the anti-cleaved NOTCH1 (NIC1) antibody while the anti-MYC antibody was used to immunoprecipate the overexpressed NIC1 (MYC-tagged). Protein G-sepharose (GE Healthcare) was subsequently added for 1h at 4°C under agitation. Immunocomplexes were washed thrice with ice-cold Triton buffer. Thereafter, immunocomplexes were either dismantled by addition of 4X Laemmli sample buffer (1X = 62.5mM Tris-HCl pH 6.8, 2.3% SDS, 10 % glycerol, 1mM PMSF, 0.005% bromophenol blue and 5% β-mercaptoethanol) or used for phosphatase and kinase assays. 

For phosphatase assays, immunocomplexes were further washed twice with 1X NEBbuffer pack for Protein MetalloPhosphatase (New England Biolabs Inc.) supplemented with 1mM MnCl_2_ and 1mM PMSF, 0.5μg/ml leupeptin, 1μg/ml pepstatin and 0.5μg/ml aprotinin. The beads were then equally split in two Eppendorf tubes. 400 units of lambda protein phosphatase (New England Biolabs Inc.) were added to one of them and both tubes were incubated for 30min at 30°C. The reaction was stopped by adding 4X Laemmli sample buffer. 

For kinase assays, Triton-washed immunocomplexes were further washed twice with ERK1 reaction buffer (25mM Tris pH 7.5, 0.02mM EGTA, 0.2mM orthovanadate, 1mM PMSF, 0.5μg/ml leupeptin, 1μg/ml pepstatin and 0.5μg/ml aprotinin). The beads were then equally split in two Eppendorf tubes and incubated 30min at 30°C in ERK1 reaction buffer supplemented with 10mM magnesium acetate, 0.1mM ATP and 2μCi [γ-^32^P]ATP containing or not 0.1μg of active ERK1 (Millipore). The reaction was stopped by adding 4X Laemmli sample buffer. Radiolabeled NIC1 was separated by SDS-PAGE and processed for autoradiography.

### Data Presentation

All experiments were performed in at least three independent experiments. Typical Western blots are shown. Densitometric analyses were performed using the Image J 1.42 software. 

## Results

### Calcium chelation activates the NOTCH1 receptor

**Figure 1:**
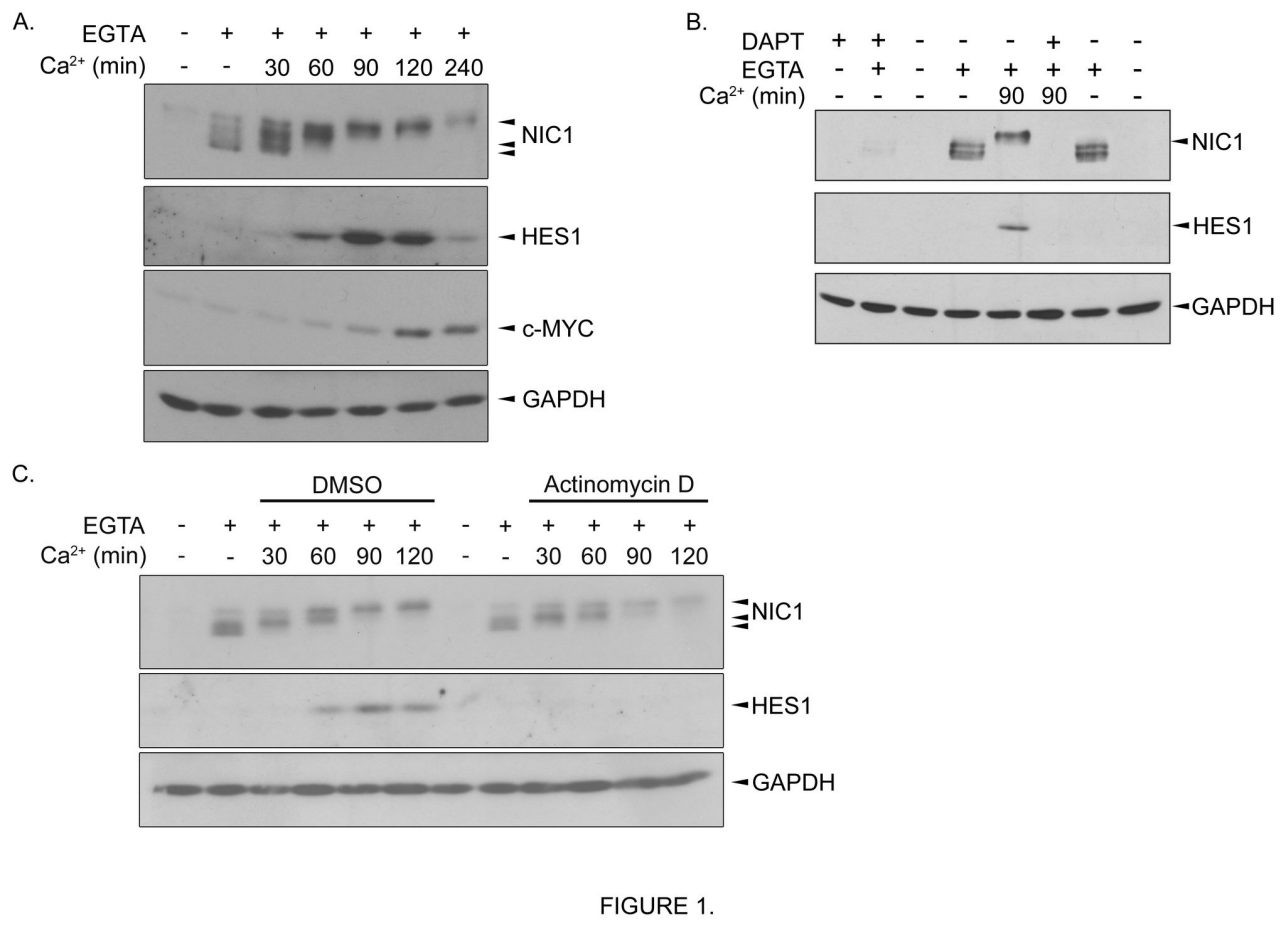
Transient exposure to EGTA leads to NOTCH1 receptor activation. **A**. MIA PaCa-2 cells were left untreated (-) or treated with EGTA (4mM) for 15min (+). EGTA-containing medium was removed and replaced by normal culture media (Ca^2+^) for the indicated time periods. **B**. MIA PaCa-2 cells were pretreated (+) or not (-) with DAPT (25μM) for 30min. Cells were then exposed to EGTA (4mM) for 15min (+). EGTA-containing medium was removed and replaced by normal culture media (Ca^2+^) containing DMSO (-) or DAPT (25μM) for 90min. C. MIA PaCa-2 cells were left untreated (-) or treated with EGTA (4mM) for 15min (+). EGTA-containing medium was removed and replaced by normal culture media (Ca^2+^) containing DMSO or actinomycin D (1μg/ml) for the indicated time periods. Western blot analyses were performed using the appropriate antibodies.

To timely study NOTCH1 activation, we exploited a model allowing the synchronous activation of the NOTCH1 receptor. Owing to the nature of the NOTCH receptors which are composed of two subunits held together non-covalently by calcium-dependent interactions [1,2], it was previously demonstrated that calcium depletion dissociates and activates the NOTCH receptors [28]. This strategy was proven a reliable method for NOTCH activation [29–34]. Therefore, we proceeded to a pulse of NOTCH activation by exposing pancreatic cancer NOTCH1-expressing cells to the calcium chelator EGTA. As expected, a 15 minutes pulse of EGTA resulted in receptor cleavage [28,31] leading to increased expression of the cleaved fragment NIC1 (Figure 1A). Following EGTA exposure, medium was removed and cells were returned for different time periods (i.e. 30 to 240 minutes) to their normal cultured condition, which contained calcium. Increased protein expression levels of NIC1 target genes HES1 and c-MYC were observed, although with slightly different temporal profile (Figure 1A). This observation is in agreement with a recent study demonstrating distinct RNA profiles of NOTCH responsive genes; members of the *E*(*spl*) family genes (the *Drosophila* homologs of the human *HES* family) being unique in the rapidity of their response to NOTCH activation [30]. 

To support NOTCH-dependent mechanisms involved in the EGTA-induced HES1 expression, we pre-treated the cells with the gamma-secretase inhibitor DAPT, well known to block NOTCH cleavage. Pre-treatment of the cells with DAPT prevented the EGTA-induced NIC1, HES1 and c-MYC protein expressions (Figure 1B and data not shown) supporting that EGTA impacts HES1 expression through activation of the NOTCH receptors. Also, addition of the transcription inhibitor actinomycin D following EGTA exposure did not significantly impact on the EGTA-induced NIC1 expression but prevented expression of HES1 and c-MYC (Figure 1C and data not shown). Taken together, our data are in line with what has been previously shown in other cell lines [29–34] i.e. transitory exposure to EGTA leads to NOTCH1 activation by releasing the intracellular domain NIC1, allowing it to translocate towards the nucleus to modulate gene transcription. Subcellular fractionation confirmed that NIC1 is predominantly found within the nucleus following EGTA exposure (Figure S1). 

### NIC1 is phosphorylated in pancreatic cancer cells

Interestingly, we detected different molecular weight forms of NIC1 following a pulse of NOTCH1 activation by EGTA with mainly high molecular weight forms gradually observed over time (Figure 1A). Noteworthy, 240 minutes after EGTA exposure, NIC1 expression returned to levels comparable to untreated cells suggesting a rapid turnover of NIC1 subsequent to its transcriptional action. Accordingly, HES1, a short-lived protein [3], was only expressed transiently, peaking at 90-120 minutes following EGTA exposure and returning about control levels after 240 minutes. 

**Figure 2:**
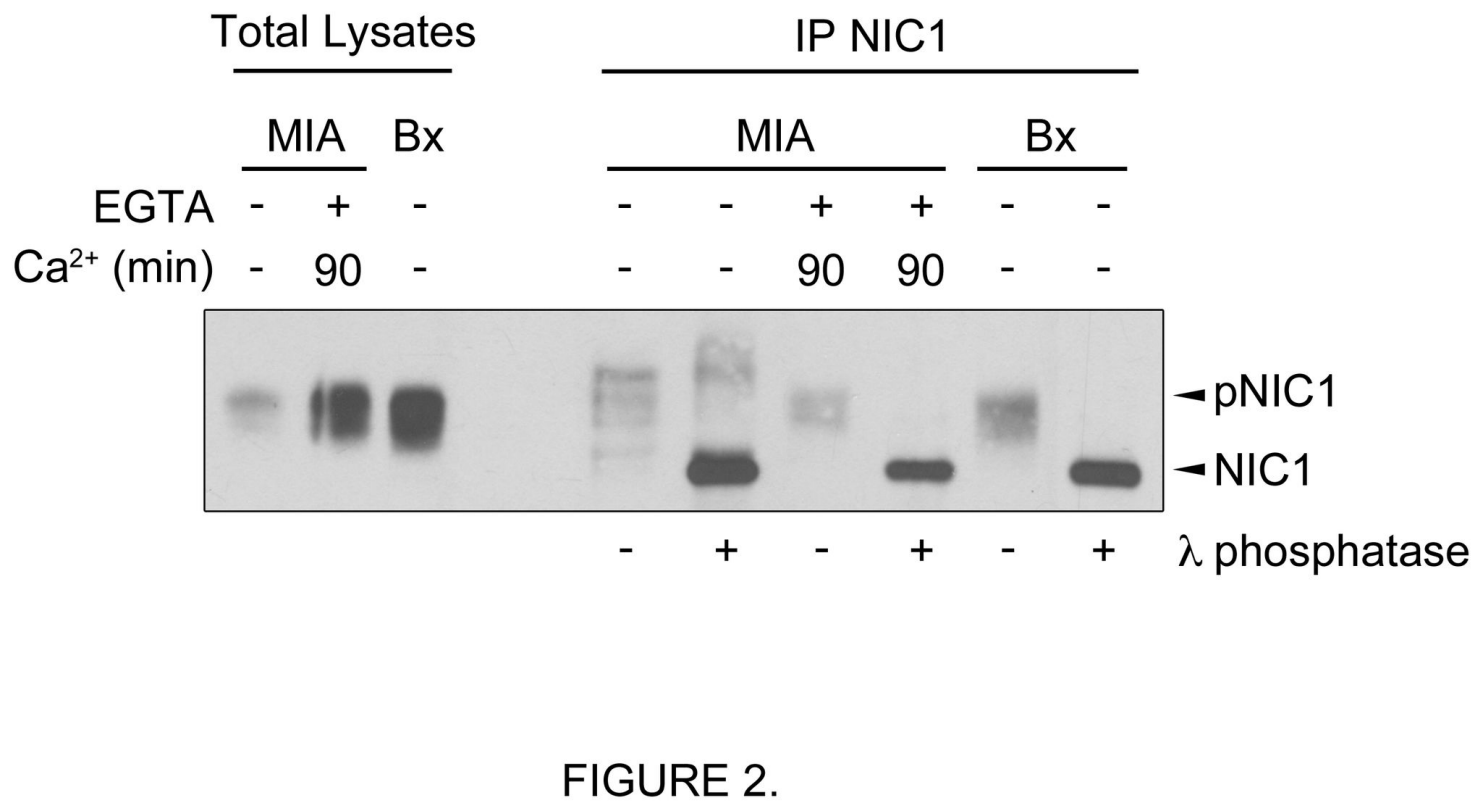
NIC1 is phosphorylated in pancreatic cancer cells. NIC1 was immunoprecipitated (IP NIC1) from total lysates of exponentially growing MIA PaCa-2 (MIA), MIA PaCa-2 cells treated with EGTA (4mM) for 15min and then returned to their normal culture media for 90min, or exponentially growing BxPC-3 (Bx). Following NIC1 immunoprecipitation, phosphatase assays were performed (+) using the lambda phosphatase (λ phosphatase). The IP NIC1 not incubated with the lambda phosphatase is indicated as pNIC1. A representative Western blot using an antibody against NIC1 is shown.

To explain the different molecular weight profile of NIC1, we tested whether NIC1 undergoes post-translational modifications following its release from the transmembrane receptor. More specifically, we analysed the phosphorylation levels of NIC1 in pancreatic cancer cells namely MIA PaCa-2 and BxPC-3. These cell lines display basal activation of the NOTCH1 receptor as denoted by the expression of the cleaved NOTCH1 fragment NIC1 (Figure 2). Noteworthy, exponentially growing MIA PaCa-2 cells display lower level of NOTCH1 activation as compared to BxPC-3. Phosphatase assays revealed that NIC1 is phosphorylated in exponentially growing MIA PaCa-2 and BxPC-3 cells (Figure 2). Moreover, ensuing EGTA-induced NOTCH1 activation in MIA PaCa-2, NIC1 was predominantly found in a phosphorylated state (Figure 2).

**Figure 3:**
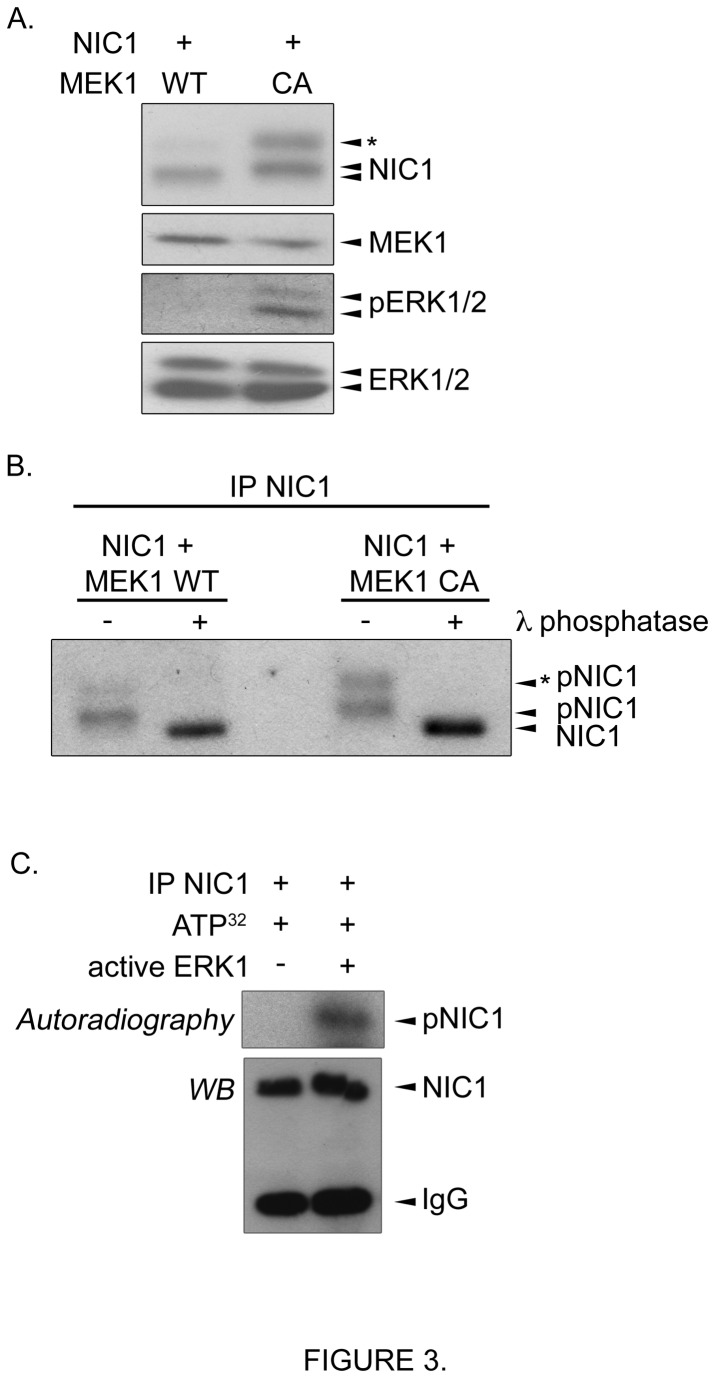
Expression of a constitutive active form of MEK1 induces NIC1 post-translational modifications when expressed in HEK293T cells. **A**. HEK293T were transfected with pLIA-NIC1 (MYC-tagged) construct together with a cDNA encoding a wild-type (WT) or constitutive active (CA) version of MEK1 (HA-tagged). Cells were lysed 24h post-transfection. NIC1 expression was analysed by western blot using an anti-MYC antibody. The expression level of the exogenous MEK1 was evaluated by western blot using an anti-HA antibody. Dual-phosphorylated and total ERK1/2 expression levels were assessed using specific antibodies. The MEK1 CA-induced higher molecular weight form of NIC1 is indicated by an asterisk *. **B**. HEK293T were transfected with pLIA-NIC1 construct together with a cDNA encoding MEK1 WT or MEK1 CA. Cells were lysed and NIC1 was immunoprecipitated (IP NIC1) using an anti-MYC antibody. Phosphatase assays were then performed (+) using the lambda phosphatase (λ phosphatase). The phosphorylated forms of NIC1 are denoted pNIC1. The MEK1 CA-induced higher molecular weight form of NIC1 is indicated by an asterisk *. NIC1 expression was analysed by western blot using an anti-MYC antibody. **C**. NIC1 was immunoprecipitated (IP NIC1) from pLIA-NIC1 transfected HEK293T using an anti-MYC antibody. Kinase assays were then performed as described in experimental procedures. The autoradiography depicting phosphorylated NIC1 (pNIC1) is shown. Following electrotransfer of the gel, the amount of NIC1 immunoprecipitated was confirmed by western blot (WB) using an anti-MYC antibody.

In our effort to identify potential kinases that modulate NIC1 phosphorylation, we proceeded to transient transfections in HEK293T cells. Interestingly, we observed higher molecular weight forms of an exogenous NIC1 when cells were co-transfected with a constitutive active form of MEK1 (MEK1 CA) that lead to ERK1/2 activation (Figure 3A). Phosphatase assays provided evidence that NIC1 is phosphorylated when expressed in HEK293T cells (Figure 3B left, NIC1 + MEK1 WT). However, NIC1 phosphorylation levels were greatly increased when the MEK/ERK pathway was activated by the presence of a MEK1 CA (Figure 3B right). Similar NIC1 phosphorylation states were detected when cells were co-transfected with an oncogenic RAS (data not shown). These results suggested that the MEK/ERK pathway could influence the phosphorylation levels of NIC1. The ERK1/2 are well known to phosphorylate a number of cytoplasmic and nuclear proteins including transcriptional regulators [35,36]. Therefore, we tested whether NIC1 could be a direct target of the ERK1/2. Interestingly, an active ERK1 was able to phosphorylate NIC1 in *in vitro* kinase assays (Figure 3C). Taken together, our results suggest that NIC1 is phosphorylated in pancreatic cancer cells. According to our observation that NIC1 phosphorylation states increased over time following EGTA exposure whilst decreasing in expression, it is tempting to speculate that NIC1 might undergo hierarchical phosphorylation events following its release from the transmembrane receptor which coordinate NIC1 expression as well as NIC1-dependent transcription activation and attenuation. Furthermore, consequent to our results, the potential involvement of the MEK/ERK pathway in the regulation of NIC1 function certainly deserves further attention.

### Activation of the MEK/ERK pathway promotes NOTCH signalling

**Figure 4:**
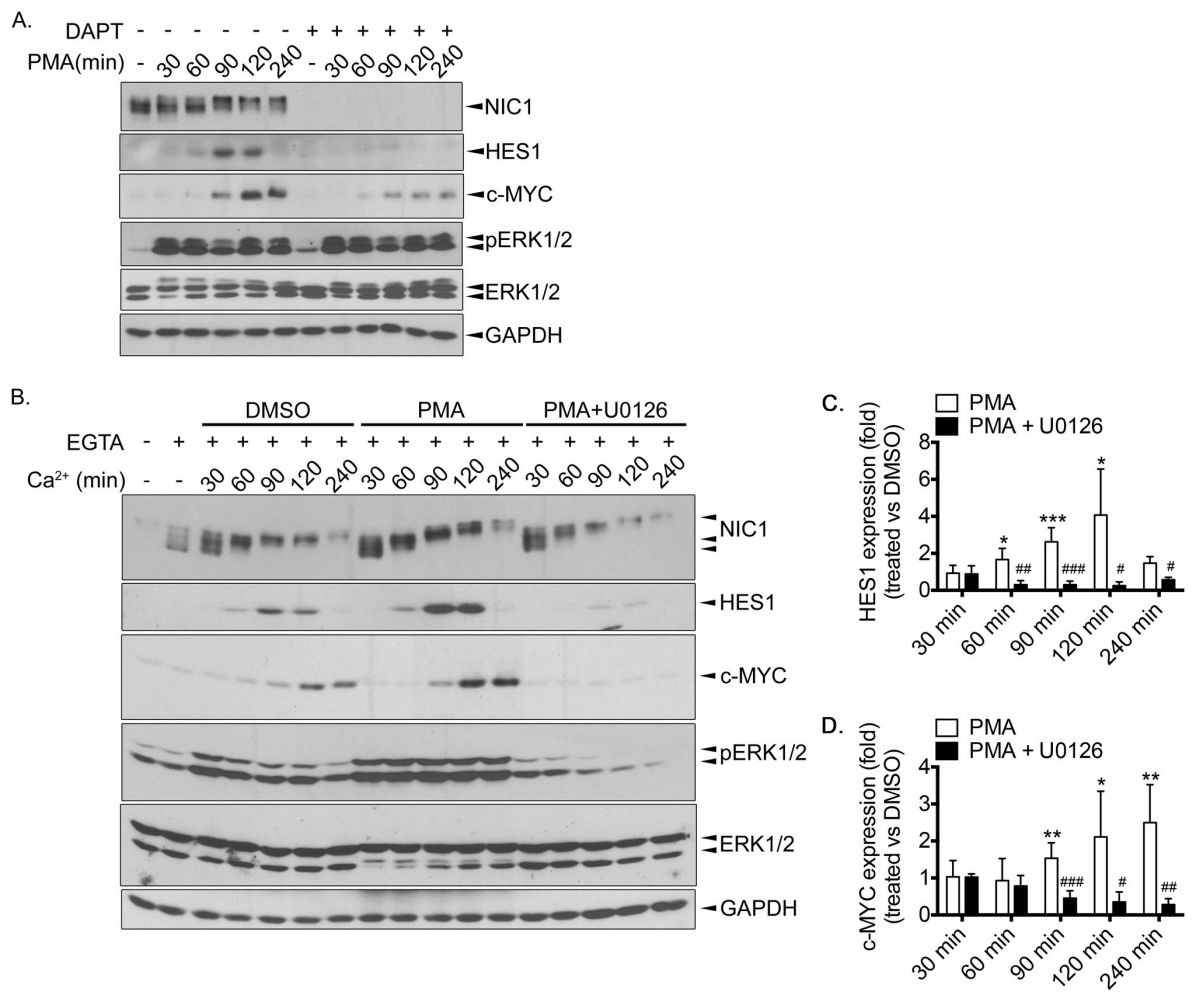
Activation of the MEK/ERK pathway promotes NOTCH signalling. **A**. MIA PaCa-2 cells were pre-treated (+) or not (-) with DAPT (25μM) overnight. Cells were then treated with PMA (100nM) for the indicated time periods. **B**. MIA PaCa-2 cells were left untreated (-) or treated with EGTA (4mM) for 15min (+). EGTA-containing medium was removed and replaced by normal culture media (Ca^2+^) containing DMSO, PMA (100nM) or PMA + U0126 (10μM) for the indicated time periods. **A**. and B. Cells were lysed and Western blot analyses were performed using the indicated antibodies. **C**. From similar experiments presented in B., a graphic representation depicting relative HES1 expression, for each time period, in treated cells (PMA, PMA+U0126) compared to control cells (DMSO-treated) is shown. **D**. From similar experiments presented in B., a graphic representation depicting relative c-MYC expression, for each time period, in treated cells (PMA, PMA+U0126) compared to control cells (DMSO-treated) is shown. **C**. and D. The results are the means ± SEM of at least three independent experiments. * p<0.05, ** p<0.005, ***p<0.0005 compared to the corresponding time period in DMSO-treated cells. # p<0.05, ## p<0.005, ### p<0.0005 compared to the corresponding time period in PMA-treated cells.

We next addressed whether the MEK/ERK pathway could influence NOTCH signalling particularly in our pancreatic cancer cell model MIA PaCa-2, which displays basal NOTCH1 activation/NIC1 expression but inducible NOTCH1 activation (see Figure 1A). Noteworthy, like most pancreatic cancer cells, MIA PaCa-2 cells harbour a KRAS mutation leading to basal ERK1/2 activation, the latter being essential for MIA PaCa-2 cell proliferation and survival [37]. To strongly and sustainably stimulate the MEK/ERK pathway, we treated MIA PaCa-2 cells with phorbol 12-myristate 13-acetate (PMA). As shown in Figure 4A, addition of PMA rapidly led to sustained ERK1/2 activation, which correlated with increased HES1 and c-MYC protein expression levels. To ensure that the effect was NOTCH-dependent, we pre-treated the cells with DAPT to drop NIC1 expression (Figure 4A) and to most likely inhibit the cleavage of the other NOTCH receptors. Pre-treatment with DAPT significantly prevented the PMA-induced HES1 expression, whereas the impact on c-MYC was partial (Figure 4A). This could potentially be explained by the fact that, although c-MYC has been identified as a NOTCH1 direct target gene [38,39], c-MYC is also known to be directly regulated by the ERK1/2 [35,36]. Therefore, it might be possible that c-MYC is regulated by both NOTCH- and ERK-dependent mechanisms in our model. Noteworthy, in our model, DAPT completely abrogated the PMA-induced HES1 expression suggesting that PMA largely promotes HES1 expression through the NOTCH receptors contrasting with previous studies suggesting a MEK-dependent NOTCH-independent regulation of HES1 [40,41]. 

To also explore the influence of ERK1/2 activation following a pulse of NOTCH1 activation, we added PMA into the media after washing out EGTA. Addition of PMA promoted the EGTA-induced HES1 and c-MYC expression, an effect that was abrogated by the presence of the MEK inhibitor U0126 (Figure 4B-D). It is worth mentioning that we denoted an increase in ERK1/2 phosphorylation that declined over time after replacing the medium of EGTA-treated cells with normal culture media (see control (DMSO-treated) cells; Figure 4B). This slight increase might represent a limitation of the method (that requires removal of EGTA) and might only be consequent of replacing the EGTA-containing media with fresh culture media, ERK1/2 phosphorylation being quite sensitive to medium change. We excluded a role for NOTCH signalling in modulating ERK1/2 activities because i) DAPT treatment prevented the EGTA-induced NIC1 expression (Figure 1B) without affecting ERK1/2 phosphorylation (data not shown) and ii) inhibition of NOTCH signalling by DAPT treatment did not significantly modulate ERK1/2 phosphorylation (Figure 4A). Taken together, our results point towards a positive regulation of NOTCH-dependent HES1 expression by the MEK/ERK pathway. 

**Figure 5:**
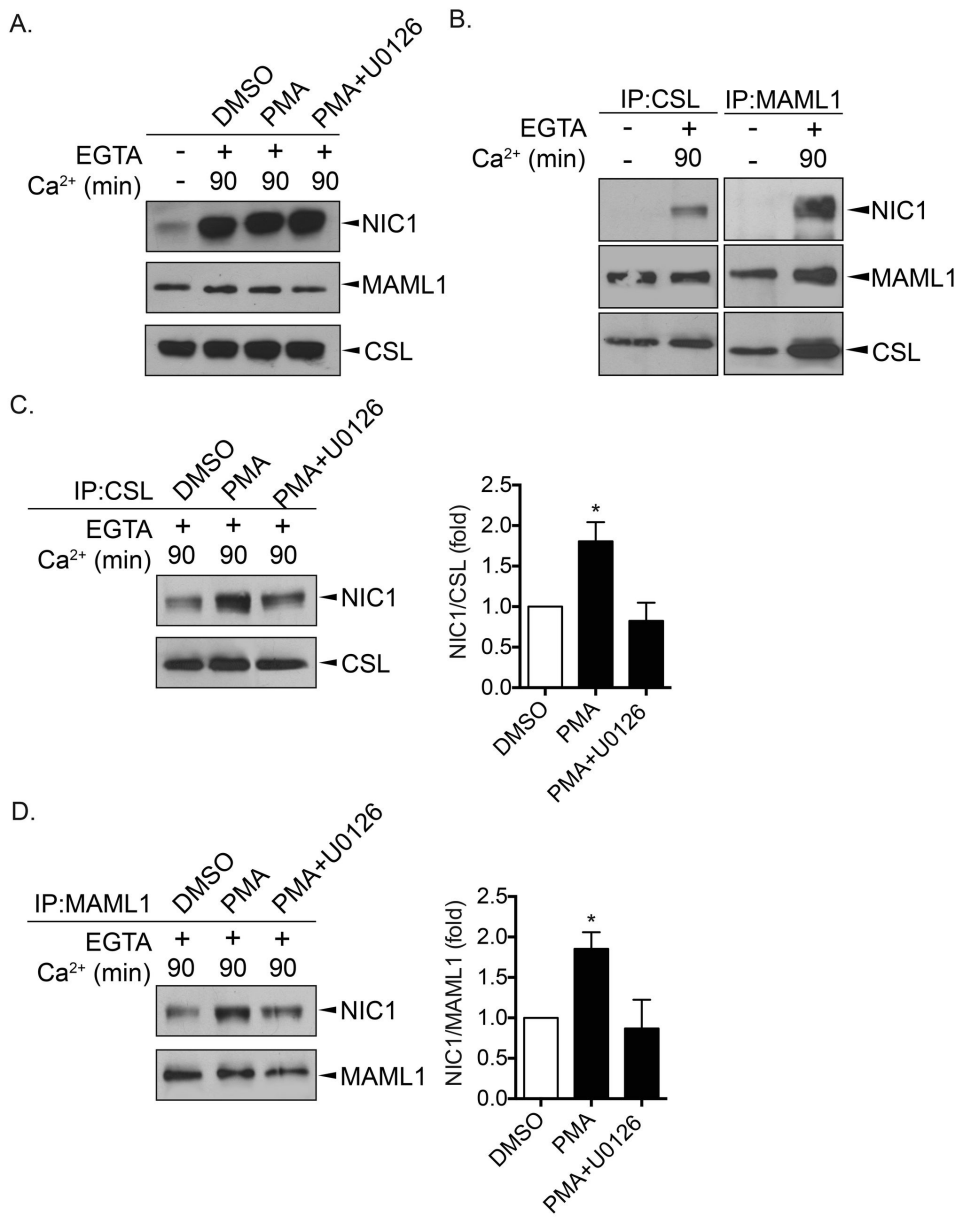
Activation of the MEK/ERK pathway promotes NIC1 association with its transcriptional partners. MIA PaCa-2 cells were left untreated (-) or treated with EGTA (4mM) for 15min (+). EGTA-containing medium was removed and replaced by normal culture media (Ca^2+^) containing DMSO, PMA (100nM) or PMA + U0126 (10μM) for 90min. A. NIC1, MAML1 and CSL expression levels were assessed by western blot using the appropriate antibodies. **B**. CSL and MAML1 were immunoprecipitated (IP) prior to western blot analyses using the indicated antibodies. **C**. CSL was immunoprecipitated (IP) followed by western blot analyses of NIC1 (left). A graphic representation depicting the relative amount of NIC1 co-immunoprecipitated with CSL (NIC1/CSL) is shown (right). **D**. MAML1 was immunoprecipitated (IP) followed by western blot analyses of NIC1 (left). A graphic representation depicting the relative amount of NIC1 co-immunoprecipitated with MAML1 (NIC1/MAML1) is shown (right). **C**. and D. The results are the means ± SEM of at least three independent experiments. * p<0.05 compared to DMSO-treated cells.

We established that the EGTA-induced HES1 expression required transcriptional events (Figure 1C) supporting a model whereby the released NIC1 translocates towards the nucleus to impact (*HES1*) gene expression. Inherent to this, the association of NIC1 with its DNA-binding partner CSL and the co-activator MAML1, hence forming a functional transcriptional unit, is certainly a prerequisite to gene modulation [1,2]. Therefore, to begin delineating the mechanisms that account for the positive impact of the MEK/ERK pathway on NOTCH signalling, we investigated whether activation of the MEK/ERK pathway could promote the association of NIC1 with either CSL or MAML1. As opposed to NIC1, CSL and MAML1 expression levels were not modulated upon EGTA treatment (Figure 5A). Consequently, we immunoprecipitated CSL and MAML1 and tested NIC1 association. Following a pulse of NOTCH1 activation, higher levels of NIC1 were found associated with either CSL or MAML1 (Figure 5B) again reinforcing our model whereby, upon EGTA-mediated NOTCH1 activation, the released NIC1 assembles into an active transcriptional complex to modulate gene expression. Interestingly, as compared to DMSO-treated cells, treatment with PMA promoted by nearly 2-fold the association of NIC1 with either CSL (Figure 5C) or MAML1 (Figure 5D). The addition of U0126 prevented both the PMA-induced NIC1/CSL and NIC1/MAML1 association (Figure 5C-D) supporting MEK-dependent mechanisms. 

## Discussion

In agreement with previous studies [29–34], our results demonstrate that a transient exposure to EGTA is a useful and reliable model to timely study NOTCH1 activation in NOTCH1-expressing cells. Indeed, it allowed us to discover that, upon release from the transmembrane receptor, NIC1 undergoes a series of post-translational modifications that include phosphorylation. It also corroborated previously established concept whereby NIC1 is a short-lived protein [1] as, within a 4h time frame, the expression of the newly cleaved NIC1 almost completely disappeared. Along with increased NIC1 expression, post-translational modifications and turnover, we were able, for the first time, to detect increased expression of endogenous NOTCH targets suggesting that the EGTA-induced NIC1 expression translates into a functional transcriptional unit in pancreatic cancer cells. The main novelty of our research is the demonstration that the MEK/ERK pathway promotes the expression of the NOTCH’s target HES1. Although more studies will definitely be needed to determine if the MEK/ERK activation impacts all or only a subset of NOTCH targets, our data provide evidence that the MEK/ERK pathway strengthens HES1 expression most likely through mechanisms dependent on the NOTCH receptors because pre-treatment of the cells with a gamma-secretase inhibitor (DAPT) prevented the PMA-induced HES1 expression. Previous studies also reported impact of the RAS pathway on NOTCH signalling [18,42]. These studies, mainly done under prolonged RAS activation or MEK inhibition, suggested that RAS signalling promotes NOTCH1 receptor cleavage/activation. Our findings are innovative since they reveal that the MEK/ERK signalling pathway is efficient to, minutes after NOTCH1 cleavage, directly influence NIC1 transcriptional function. One hypothesis arising from our results is that the MEK/ERK pathway promotes the assembly of a functional NIC1 transcriptional unit with CSL and MAML1. However, further studies are required to delineate the precise mechanisms by which the MEK/ERK pathway promotes NOTCH signalling.

Interestingly, it was previously suggested that post-translational modifications, particularly phosphorylation, is almost certainly modulating NIC1-dependent transcription [1]. Initially in *Drosophila* [43], but later on in mammalian, hyperphosphorylation of the intracellular domain of NOTCH1 and NOTCH2 was detected following their release from the transmembrane receptor [44,45]. Noteworthy, hyperphosphorylation of NIC1 was associated with i) NIC1 nuclear accumulation [45–48], ii) NIC1 interaction with CSL [44], iii) increased CSL-dependent transcription [44,47,48] and iv) NIC1 transforming capacity [48]. These results suggested that NIC1 phosphorylation might promote the transcriptional potential of the NIC1/CSL complex. However, to date, mostly kinases that negatively regulate NIC1 function have been identified: AKT was shown to promote NIC1 cytoplasmic localization [49], phosphorylation of NIC1 by DYRK1A attenuated NOTCH signalling [50], and NLK-dependent NIC1 phosphorylation decreased the formation of the NIC1/CSL complex [51]. Capobianco group recently provided evidence that prior or during NIC1/CSL/MAML1 complex association, NIC1 is phosphorylated by CK2 allowing the accessibility to a subsequent phosphorylation site [52]. Both phosphorylation events appeared required for dissociation of the complex from DNA. In addition, the CycC/CDK8 complex is thought to play a major role in disrupting the NIC1/CSL/MAML1 complex by phosphorylating NIC1 within its C-terminal PEST domain ensuing FBW7-mediated NIC1 ubiquitination and degradation [53]. Nevertheless, to date, no positive NIC1 regulators (kinases) were identified and nearly all phosphorylation sites on NIC1 remain to be determined. Our results now provide the basis for investigating the positive contribution of the MEK/ERK pathway on NOTCH signalling particularly in the context of pancreatic cancer cells. Noteworthy, it was previously shown that the forced expression of NIC1 in the mouse pancreatic epithelium promotes the formation of premalignant pancreatic lesions initiated by an oncogenic *KRAS* [54] suggesting that KRAS and NOTCH signalling cooperate to promote pancreatic carcinogenesis. The mechanisms underlying this cooperation were not investigated and it remains to be determined whether this cooperation requires activation of the MEK/ERK pathway. Interestingly, by using RAS effector loop mutants that bind to, and specifically activate only one of the pathway effectors, it was demonstrated in breast cancer cells that NIC1 only cooperates with the RAF-activating RAS mutant for the promotion of anchorage-independent growth [55]. It remains to be addressed whether the RAS-NOTCH cooperation observed in pancreatic epithelial cells and other models also operates through the MEK/ERK pathway. Still, for the first time, our results uncovered a potential mechanism of action that could account for RAS-NOTCH cooperation in carcinogenesis [17,54,55] viz. the MEK/ERK pathway promoting expression of NOTCH target genes thereby influencing NOTCH signal strength. This could be particularly relevant since dose dependent differences in the NOTCH response have been observed in mammary epithelial cells [56].

## Supporting Information

Figure S1 **Nuclear localization of NIC1 upon EGTA exposure.** MIA PaCa-2 cells were left untreated (-) or treated with EGTA (4mM) for 15min (+). EGTA-containing medium was removed and replaced by normal culture media (Ca^2+^) for 90min before proceeding to nuclear/cytosolic fractionation. Proteins obtained from cytosolic (cytoplasm) and nuclear extracts were separated on SDS-PAGE followed by western blot analyses of NIC1. LAMIN B represents a control protein for the nuclear fraction.
(PDF)
