# Supplementary material for: The MEK/ERK Pathway Promotes NOTCH Signalling in Pancreatic Cancer Cells
Source: PLoS One. 2013 Dec 31;8(12):e85502. doi: 10.1371/journal.pone.0085502 (PMC3877363; doi:10.1371/journal.pone.0085502)
Supplement: Figure S1 — Nuclear localization of NIC1 upon EGTA exposure. MIA PaCa-2 cells were left untreated (-) or treated with EGTA (4mM) for 15min (+). EGTA-containing medium was removed and replaced by normal culture media (Ca2+) for 90min before proceeding to nuclear/cytosolic fractionation. Proteins obtained from cytosolic (cytoplasm) and nuclear extracts were separated on SDS-PAGE followed by western blot analyses of NIC1. LAMIN B represents a control protein for the nuclear fraction. (PDF) [file pone.0085502.s001.pdf]

SUPPLEMENTARY INFORMATION

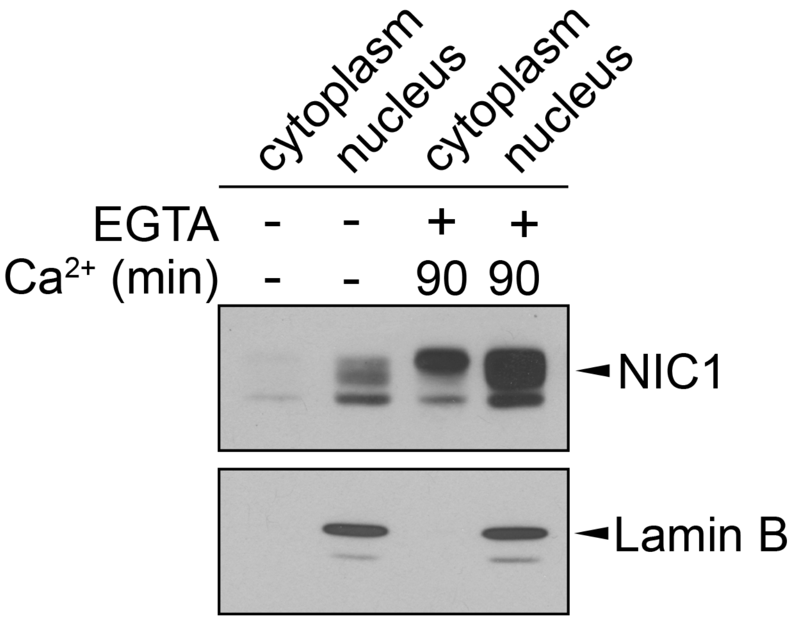

FIGURE S1. Pulse of NOTCH1 activation leads to NIC1 nuclear localization.
MIA PaCa-2 cells were left untreated (-) or treated with EGTA (4mM) for 15min (+). EGTA-containing medium was removed and replaced by normal culture media (Ca^{2+}) for 90min. Nuclear and cytoplasmic extracts were prepared and separated on SDS-PAGE. NIC1 expression levels in cytoplasmic and nuclear extracts were analysed by western blotting. As a control for nuclear protein, Lamin B expression levels were assessed.
